# Chinese Expert Consensus on ovarian function and fertility preservation of cervical cancer in pregnancy (2023)

**DOI:** 10.3389/fendo.2023.1280631

**Published:** 2023-12-13

**Authors:** Xiangyan Ruan

**Affiliations:** ^1^ Department of Gynecological Endocrinology, Beijing Obstetrics and Gynecology Hospital, Capital Medical University, Beijing Maternal and Child Health Care Hospital, Beijing, China; ^2^ Department of Women’s Health, University of Tuebingen, University Women’s Hospital and Research Centre for Women’s Health, Tuebingen, Germany

**Keywords:** cervical cancer in pregnancy, ovarian function, fertility preservation, expert consensus, premature ovarian insufficiency

## Abstract

Cervical cancer in pregnancy (CCIP) refers to cervical cancer diagnosed during pregnancy, the most common gynecological malignant tumor. Because of the special physiological changes of CCIP, although preserving ovarian function and fertility is very important, the methods are very limited. There is no guideline or consensus on the preservation methods of ovarian function and fertility in this special period. Therefore, the Committee of Fertility Protection and Preservation of China Association for the Promotion of Health Science and Technology, combined with the Chinese Society of Gynecological Endocrinology affiliated to the International Society of Gynecological Endocrinology, Society Endocrinology Branch of Beijing Institute of Obstetrics & Gynecology, combined with Society on Fertility Preservation affiliated with the Chinese Preventive Medicine Association, organized relevant experts from different disciplines to formulate this consensus, in order to guide ovarian function and fertility preservation of CCIP patients.

## 1 Introduction

Cervical cancer in pregnancy (CCIP) is the most common gynecological malignant tumor diagnosed during pregnancy, accounting for 71.6% (1, 2). The median age of CCIP diagnosis is about 35 years old, and the most common pathological type is squamous cell carcinoma, with a median gestational age of 18.4 weeks (3–5). Because CCIP patients are all women of reproductive age, pelvic radiotherapy, and neoadjuvant chemotherapy will seriously damage their ovarian function, and preserving ovarian function and fertility is necessary.

However, due to the particularity of CCIP patients during pregnancy, under the action of high estrogen and progesterone during pregnancy, the hypothalamus is strongly negatively fed back, which inhibits the synthesis and secretion of follicle-stimulating hormone (FSH) and luteinizing hormone (LH), and the follicles in the ovary are in the state of primordial or primary follicles, so ovarian stimulation cannot be carried out. Even after the termination of pregnancy, stimulation from the primordial or primary follicular state to mature follicles takes longer than non-pregnancy. In addition, CCIP patients can take delayed treatment when their condition is stable, and cervical cancer treatment, including surgery, radiotherapy, and chemotherapy, should be started as soon as possible when pregnancy is terminated or after delivery. These treatments, especially radiotherapy and chemotherapy, may lead to serious damage to ovarian function, premature ovarian failure, fertility loss, and various chronic diseases, seriously affecting patients’ quality of life and reproductive health (6, 7).

Because of the special physiological changes of CCIP, although preserving ovarian function and fertility is very important, the methods are very limited. There is no guideline or consensus on the preservation methods of ovarian function and fertility in this special period. However, due to the particularity of younger incidence of cervical cancer in China, some CCIP patients still face the special needs of ovarian function and fertility preservation. Therefore, the Committee of Fertility Protection and Preservation of China Association for the Promotion of Health Science and Technology, combined with the Chinese Society of Gynecological Endocrinology affiliated to the International Society of Gynecological Endocrinology, Society Endocrinology Branch of Beijing Institute of Obstetrics & Gynecology, combined with Society on Fertility Preservation affiliated with the Chinese Preventive Medicine Association, organized relevant experts from different disciplines to formulate this consensus, in order to guide ovarian function and fertility preservation of CCIP patients.

See **Table 1** for the recommended level and representative significance of this consensus.

**Table 1:** Recommendation level of this consensus and its representative significance.

Recommended level	Representative meaning
Category 1	Based on high-level clinical research evidence, expert opinions are highly consistent.
Category 2A	Based on the evidence of low-level clinical research, experts’ opinions are highly consistent; Or based on high-level evidence, experts’ opinions are consistent.
Category 2B	Experts’ opinions are consistent based on the evidence of low-level clinical research.
Category 3	No matter what level of clinical evidence is based on, experts’ opinions are different.

## 2 Multidisciplinary diagnosis and treatment in CCIP

Cervical cancer screening should be carried out according to the standard during pregnancy. The diagnosis and staging of CCIP are similar to those of non-pregnancy, and the clinical staging of CCIP is adopted by the International Federation of Gynecology and Obstetrics (FIGO), which should be differentiated from other diseases before diagnosis (8). Magnetic resonance imaging (MRI) with sufficient sensitivity and specificity is recommended for CCIP disease evaluation to provide lesion infiltration range, parametrial diffusion, and lymph node metastasis, which is helpful to make up for the dissatisfaction of gynecological examination during pregnancy and the lack of ultrasound examination (9).

The treatment of CCIP is more complex and challenging. Due to the lack of prospective studies and clinical trials and the lack of standardized procedures, it needs to be individualized, taking into account the patient’s age, tumor size, disease stage, histopathological type, lymph node involvement, pregnancy weeks, fetal intrauterine condition, the willingness of patients and their families to continue pregnancy, the desire to give birth in the future, and the advantages and disadvantages of various treatment methods. Obstetrics, neonatology, gynecological oncology, pathology, imaging, and other disciplines need to participate in formulating treatment plans. Strike a balance between effective treatment of tumors and protection of fetal health to avoid delayed treatment and premature delivery (10, 11). For details, please refer to the Chinese Expert Consensus on Diagnosis and Treatment of Pregnancy Complicated with Cervical Cancer, formulated by the Chinese Obstetricians and Gynecologists Association (COGA) (11).

Pregnancy itself may not have a negative impact on the prognosis of cervical cancer, but if the pregnancy continues and treatment is delayed, it will affect the maternal prognosis to a certain extent (5, 12, 13). In the first trimester of pregnancy, it takes a long time for the fetus to mature, which has a high risk of disease progression. Patients are generally not recommended to continue pregnancy, and they should receive routine cervical cancer treatment as soon as possible (5). For patients with early cervical cancer, delaying treatment to achieve fetal survival or improve fetal outcomes may be an option (14). For patients with 14 ~ 20 weeks of pregnancy, FIGO 2018 IB1 or IIA, neoadjuvant chemotherapy can be used until fetal maturity. The chemotherapy scheme is still managed regarding non-pregnancy chemotherapy guidelines. The platinum-based combined scheme is preferred, but chemotherapy is avoided 3 ~ 4 weeks before delivery to reduce bone marrow suppression of mother and child caused by chemotherapy (15, 16). The treatment can be postponed for 20 ~ 33 weeks of pregnancy until a cesarean section is performed to promote fetal lung maturation. For IB1 or above, neoadjuvant chemotherapy can be selected. The initial treatment time of delayed treatment should not exceed 32 ~ 34 weeks of pregnancy, and pregnancy can be terminated after fetal lung maturation (14). CCIP patients suggest a cesarean section to terminate the pregnancy, and cervical cancer should be treated routinely after delivery (13). The prognosis of CCIP patients is similar to that of non-pregnancy, related to FIGO staging and treatment methods (17).

**Recommendation:** CCIP’s ovarian function and fertility preservation need multidisciplinary cooperation, combined with factors such as tumor stage, gestational age, and patient’s pregnancy willingness, and choosing full communication between doctors and patients (recommended level 2A).

## 3 Ovarian function and fertility preservation strategy in CCIP

According to the National Cancer Institute of the National Institutes of Health data, the 5-year relative survival rate of patients with early cervical cancer is about 91%, and that of all patients with cervical cancer is 67%. However, surgery, radiotherapy, and chemotherapy will lead to ovarian insufficiency or failure, leading to early climacteric symptoms in young women, such as osteoporosis, cardiovascular and cerebrovascular diseases, and other chronic diseases, and the risk of death will increase significantly (8). The formulation of this consensus draws lessons from and strictly refers to the indications of ovarian preservation in cervical cancer (18, 19). On the premise of not affecting the survival outcome of patients with tumors, it is suggested that CCIP patients who meet the conditions of ovarian preservation should be protected in the diagnosis and treatment process to prevent the occurrence of iatrogenic premature ovarian insufficiency (POI). Because CCIP patients cannot perform ovarian stimulation and embryo and oocyte cryopreservation, this consensus only involves ovarian tissue cryopreservation and transplantation (OTCT) and ovarian transposition in CCIP patients.

### 3.1 Ovarian tissue cryopreservation and transplantation technology

#### 3.1.1 Overview

OTCT technology includes removing part of ovarian tissue before gonadal toxicity treatment (including radiotherapy and chemotherapy), carrying out programmed freezing by cryobiology technology, and then cryopreservation at ultra-low temperatures. When patients need it and conditions permit, the cryopreserved ovarian tissue will be thawed and transplanted back into the body (20). In the 1860s, ovarian tissue was first cryopreserved and transplanted in rodents (21). In 1994, ovarian tissue was cryopreserved and transplanted in sheep (22). In 1996, Hovatta et al. successfully cryopreserved human ovarian tissue (23), and in 1998, the first xenogeneic model showed the feasibility of frozen-thawed human ovarian tissue transplantation (24). In 2000, the first human cryopreserved-thawed ovarian tissue was successfully transplanted *in situ* (25). The first human ovarian cortex was successfully heterotopic transplanted in 2001 (26), the first baby was born after human cryopreserved-thawed ovarian tissue transplantation in 2004, and the second baby was born in 2005 (27, 28). Our team successfully carried out the first Chinese orthotopic transplantation of human frozen-thawed ovarian tissue in 2016 (29), the first pregnancy after frozen-thawed ovarian tissue transplantation in 2020 (30), and the first healthy baby was born after frozen-thawed ovarian tissue transplantation in 2021 (31).

OTCT technology conforms to The European Society of Human Reproduction and Embryology (ESHRE), European Society for Medical Oncology (ESMO), American Society for Reproductive Medicine (ASRM), and French guidelines. It is a standard fertility and ovarian function preservation method and the only one for prepuberty girls and young women of reproductive age whose gonadal toxicity treatment cannot be delayed (20, 32–35). Over 200 healthy babies have been born by OTCT technology globally (36). However, only over 20 mature centers are globally developing OTCT technology (37). More than 500 cases of human ovarian tissue have been successfully cryopreserved in China’s first ovarian tissue cryobank, of which 31.6% are patients with cervical cancer, including three patients with CCIP, all of whom were cryopreserved at the same time when cesarean section terminated the pregnancy. Currently, 19 cases have been successfully transplanted in this center, and the success rate of transplantation is 100%, far exceeding the international average level (70%) (38–40).

#### 3.1.2 Application of OTCT in CCIP patients

Because CCIP patients who receive neoadjuvant chemotherapy cannot effectively superovulation, OTCT can be selected at this time (41). Without additional anesthesia and surgery, ovarian tissue biopsy can be performed during lymph node resection or cesarean section (42). The screening criteria for OTCT in CCIP patients can refer to the Chinese Expert Consensus on Ovarian Tissue Cryopreservation and Transplantation and Group Standard on Technical Specifications for Ovarian Tissue Cryopreservation and Transplantation (20, 37).

##### 3.1.2.1 Ovarian tissue biopsy

Ovarian tissue biopsy should avoid corpus luteum as much as possible, use a cold knife, and take more than 1/2 of the volume of one or both ovaries (the amount should be determined individually according to the patient’s condition). Energy instruments should not be used to contact the ovaries to avoid thermal damage, and the integrity of the taken ovarian tissue should be ensured as much as possible (37).

##### 3.1.2.2 Ovarian tissue transport

The ovarian tissue removed by surgery should be immediately put into an aseptic transfer fluid, and a special transfer box should be used to maintain a low temperature (4°C ~ 8°C) and transfer to the ovarian tissue cryobank. The transport time should not exceed 24 hours. In order to achieve standard quality control, optimize patient management, and cost-effectiveness, organizational acquisition can be carried out locally. The cryopreservation and storage of ovarian tissue should be centralized (37).

##### 3.1.2.3 Ovarian tissue cryopreservation

The ovarian tissue preparation, cryopreservation, and storage shall be performed in a laboratory meeting the requirements. When ovarian tissue is prepared, a sterile scalpel and forceps should be used to carefully remove the medulla and preserve the intact cortex. After preparation, the thickness of ovarian tissue should be about 1mm, and the size of each piece should be about 4mm × 8mm. After treatment, the ovarian tissue slices should be immediately put into cryopreservation solution for pre-cooling and balance for 20 min and then put into cryotubes containing cryopreservation solution to start freezing. Slow freezing is controlled by the computer program so that ovarian tissue is cooled to -140°C in stages according to the set rate, and then the cryopreservation tube is stored in liquid nitrogen at -196°C. Each cryopreservation tube should be marked with the patient’s name, date of birth, and code, record the storage location, and put in a liquid nitrogen tank (37).

##### 3.1.2.4 Ovarian tissue transplantation

Ovarian tissue transplantation can be divided into orthotopic transplantation (pelvic cavity) and ectopic transplantation (extrapelvic cavity). Ovarian tissue transplantation should choose orthotopic transplantation, and ectopic transplantation can be considered if orthotopic transplantation cannot be carried out for various reasons. Orthotopic transplantation can occur in the original ovary, peritoneal bag, etc. It is advisable to make an incision where the peritoneal blood supply of the lateral wall of the ovary is good, make a peritoneal bag, and put and suture the resuscitated ovarian tissue piece (37).

Concerning the safety of ovarian tissue transplantation in patients with cervical cancer, the risk factors related to ovarian metastasis include age, FIGO stage, tumor size, pathological type (such as squamous cell carcinoma or adenocarcinoma), deep stroma, uterine cavity, endometrium, vagina, blood vessels and lymph nodes infiltration (43). Ovarian preservation is safe in young patients with early cervical cancer and has no significant effect on overall or progression-free survival (44, 45). The most common pathological type of CCIP is squamous cell carcinoma, and the ovarian metastasis rate is low (4). No tumor recurrence has been related to OTCT globally (36). For patients with severe climacteric symptoms before and after transplantation of cryopreserved ovarian tissue, traditional Chinese medicine is widely used as one of the drug therapies for climacteric syndrome. According to clinical syndromes, rational drug use (such as Kuntai Capsule) can improve menopause-related symptoms of POI (46).

##### 3.1.2.5 Transplantation outcome

Most ovarian tissues recovered their ovarian function 3~4 months after transplantation, and the average maintenance time was 4~5 years (45). The ovarian function of the first cervical cancer patient with cryopreserved ovarian tissue transplantation in China has been maintained for more than seven years (29, 40), and it could be transplanted many times. The largest ovarian tissue cryobank in China reported that nine patients with cervical cancer had undergone cryopreserved ovarian tissue transplantation, and the ovarian function was restored after transplantation (20, 38). The age, ovarian reserve, and exposure to gonadal toxicity before cryopreservation of ovarian tissue are very important to the recovery of fertility, endocrine function, and ovarian functional life (47). Theoretically, when the function of the previous ovarian tissue begins to weaken, implanting another new cryopreserved ovarian tissue will avoid the simultaneous depletion of the reserves of multiple ovarian tissues and theoretically prolong the survival time of cryopreserved ovarian tissue and the maintenance time of estrogen and progesterone endocrine function (48).

### 3.2 Ovarian transposition

In recent years, the treatment of CCIP has gradually changed from active to more conservative treatment, especially for patients with early cervical cancer in the second and third trimesters of pregnancy (49). Radical resection of cervical cancer and postoperative selective pelvic radiotherapy with/without platinum drugs are the main methods to treat cervical cancer (50). In 2021, the Professional Committee of Gynecological Oncology (Study Group) of Micro-non-invasive Medicine Professional Committee of the Chinese Medical Doctor Association put forward the consensus of Chinese experts on fertility-preserving surgery for early cervical cancer (51). If combined with pelvic radiotherapy, the ovary can be moved to the planned irradiation field to protect the endocrine and reproductive function of the ovary. Ovarian transposition and shielding are recommended for cervical cancer patients under 40 (52), and the effective rate for women under 40 is about 20% ~ 90% (53). The success rate depends on the patient’s age, radiotherapy dose, transposition position, surgical skills, and whether chemotherapy is performed simultaneously (54). At present, some studies believe that the transposition of the ovary itself will also lead to the reduction of ovarian reserve (55), and it is necessary to conduct a prospective study on the long-term ovarian function of cervical cancer patients who receive ovarian transposition. Ovarian transposition and OTCT may be necessary for successful pregnancy (54, 56). Only ovarian transposition cannot protect against the adverse effects caused by chemotherapy (35).

**Recommendation:** Once CCIP patients are diagnosed, they need counseling on ovarian function and fertility preservation. OTCT technology does not delay the treatment of CCIP patients, which can preserve the fertility potential and the ovarian endocrine function of patients. Ovarian transposition alone is not recommended to protect patients’ ovarian function (recommended level: Class 2A).

## 4 Summary

With the development of cancer diagnosis and treatment technology, more and more attention has been paid to cancer survivors’ quality of life and fertility. Therefore, it is advocated to establish a multidisciplinary joint management mechanism for fertility preservation and protection of female cancer patients in all medical institutions that carry out cancer treatment and to implement a joint cancer fertility care plan in centers that treat young cancer patients so that once young women are diagnosed with cancer, they and their families can immediately get counseling on fertility preservation. Screening and evaluation of CCIP before and during pregnancy, through multidisciplinary team cooperation, will provide counseling for patients to maximize maternal and infant outcomes; those with strict indications can carry out OTCT and ovarian transposition.

## First author and correspondence author

Xiangyan Ruan (E-mail: ruanxiangyan@ccmu.edu.cn, Beijing Obstetrics and Gynecology Hospital, Capital Medical University. Beijing Maternal and Child Health Care Hospital).

## Author contributions

RX: Conceptualization, Funding acquisition, Supervision, Writing – review & editing.

## Other experts involved in the consensus

Juan Du, Jiaojiao Cheng, Fengyu Jin, Muqing Gu, Yanglu Li, Yinmei Dai, Yumei Wu, Weimin Kong, Liying Zou, Li Zhou, Rui Ju, Chanwei Jia, Chenghong Yin, Alfred O. Mueck (Beijing Obstetrics and Gynecology Hospital, Capital Medical University. Beijing Maternal and Child Health Care Hospital); Liangzhi Xu (West China Second University Hospital, Sichuan University); Mulan Ren (Zhongnan Hospital Southeast University); Ruifang Wu (Peking Univercity Shenzhen Hospital); Xin Yang (Peking University People’s Hospital); Rong Chen (Peking Union Medical College Hospital, Peking Union Medical College/Chinese Academy of Medical Sciences); Aizhen Zhu (Yuncheng Central Hospital); Wei Ma (Beijing Luhe Hospital, Capital Medical University); Aimei He (Fuqing City Hospital of Fujian); Fenge Huang (The Fifth Hospital of Cangzhou City, Hebei Province); Hongyan Sun (Shi jiazhuang TCM Hospital); Qianqing Wang (Xinxiang Central Hospital); Xin Du (Maternal and Child Health Hospital of Hubei Province); Lihua Lin (The Affiliated Hospital of Putian University); Ying Ye (Shangqiu First People’s Hospital); Songyun Han (Tongzhou Maternal and Child Health Hospital of Beijing); Chanyu Zhang (The Second Affiliated Hospital of Chongqing Medical University); Nuo Yi (Beijing Ditan Hospital, Capital Medical University); Lei Zhang (Beijing Tsinghua Changgung Hospital, School of Clinical Medicine, Tsinghua University); Lan Nie (Hunan Maternal and Child Health Hospital); Youguo Chen (The First Affiliated Hospital of Soochow University); Hua Zheng (Beijing Chaoyang District Maternal and Child Health Hospital); Chun Zhang (The Central Hospital of Wuhan); Junhua Bao (Changhai Hospital, Naval Medical University); Yanhong Shi (Beijing Chaoyang District Taiyanggong Community Health Service Center); Ruiying Li (Beijing Fengtai District Maternal and Child Health Hospital); Pengming Sun (Fujian Maternity and Child Health Hospital, College of Clinical Medicine for Obstetrics & Gynecology and Pediatrics, Fujian Medical University); Yamei Hou (The Fifth People’s Hospital in Hengshui); Yun Liu (Beijing Friendship Hospital Affiliated to Capital Medical University); Jian Gao (Hebei General Hospital); Yan Liu (Puren Hospital Affiliated to Wuhan University of Science and Technology); Liping Cai (First Affiliated Hospital of Nanchang University); Xiaodan Zhao (XingTai Infertility Specialized Hospital).
